# Enhancement in the Structural, Electrical, Optical, and Photocatalytic Properties of La_2_O_3_-Doped ZnO Nanostructures

**DOI:** 10.3390/ma15196866

**Published:** 2022-10-02

**Authors:** Vanga Ganesh, Thekrayat H. AlAbdulaal, Manal AlShadidi, Mai S. A. Hussien, Abdelfatteh Bouzidi, Hamed Algarni, Heba Y. Zahran, Mohamed Sh. Abdel-wahab, Mervat I. Mohammed, Ibrahim S. Yahia, Bayapa Reddy Narapureddy

**Affiliations:** 1 Laboratory of Nano-Smart Materials for Science and Technology (LNSMST), Department of Physics, Faculty of Science, King Khalid University, P.O. Box 9004, Abha 61413, Saudi Arabia; 2 Research Center for Advanced Materials Science (RCAMS), King Khalid University, P.O. Box 9004, Abha 61413, Saudi Arabia; 3 Department of Chemistry, Faculty of Education, Ain Shams University, Roxy, Cairo 11757, Egypt; 4 Nanoscience Laboratory for Environmental and Bio-Medical Applications (NLEBA), Semiconductor Lab., Metallurgical Lab.1, Department of Physics, Faculty of Education, Ain Shams University, Roxy, Cairo 11757, Egypt; 5 Research Unit, Physics of Insulating and Semi-Insulating Materials, Faculty of Sciences, University of Sfax, P.O. Box 1171, Sfax 3000, Tunisia; 6 Preparatory Year Program, Shaqra University, Al-Quwayiyah Branch, Shaqra 19248, Saudi Arabia; 7 Materials Science and Nanotechnology Department, Faculty of Postgraduate Studies for Advanced Sciences, Beni-Suef University, Beni Suef 62511, Egypt; 8 Department of Public Health, College of Applied Medical Sciences, King Khalid University, Asir Region, P.O. Box 61421, Abha 61413, Saudi Arabia

**Keywords:** combustion method, La_2_O_3_-ZnO nanostructured composites, structural properties, optical band gap, electrical conductivity/electrical properties, varistors, photocatalytic activity

## Abstract

A lanthanum oxide (La_2_O_3_)-ZnO nanostructured material was synthesized in the proposed study with different La_2_O_3_ concentrations, 0.001 g to 5 g (named So to S7), using the combustion method. X-ray diffraction (XRD), scanning electron microscopy (SEM), and Fourier transformation infrared spectroscopy (FT-IR) were utilized for investigating the structure, morphology, and spectral studies of the La_2_O_3_- ZnO nanomaterials, respectively. The results obtained from previous techniques support ZnO’s growth from crystalline to nanoparticles’ fine structure by changing the concentrations of lanthanum oxide (La_2_O_3_) dopants in the host matrix. The percentage of ZnO doped with La- influences the ZnO photocatalytic activity. SEM analysis confirmed the grain size ranged between 81 and 138 nm. Furthermore, UV-Vis diffuse reflectance spectroscopy was performed to verify the effects of La_2_O_3_ dopants on the linear optical properties of the nano-composite oxides. There was a variation in the energy bandgaps of La_2_O_3_-ZnO nanocomposites, increasing the weight concentrations of lanthanum dopants. The AC electrical conductivity, dielectric properties, and current–voltage properties support the enactment of the electrical characteristics of the ZnO nanoparticles by adding La_2_O_3_. All the samples under investigation were used for photodegradation with Rhodamine B (RhB) and Methylene Blue (MB). In less than 30 min of visible light irradiation, S4 (0.5 g) La_2_O_3_-ZnO reached 99% of RhB and MB degradation activity. This study showed the best photocatalytic effect for RhB and MB degradation of 0.13 and 0.11 min^−1^ by 0.5 g La_2_O_3_-ZnO. Recycling was performed five times for the nanocatalysts that displayed up to 98 percent catalytic efficiency for RhB and MB degradation in 30 min. The prepared La_2_O_3_-ZnO nanostructured composites are considered novel candidates for various applications in biomedical and photocatalytic studies.

## 1. Introduction

For several decades, considerable research has focused on studying the argent environmental problem of water pollution [1]. To illustrate, volatile organic compounds include widespread pollution sources, primarily sewage, industrial, farming, and wastewater treatment. In nanomaterials, metal oxides demonstrate excellent properties such as being semiconductors and insulators [2,3]. Recently, nanostructured zinc oxide (ZnO) has gained intensive interest due to structural, electrical, optical, and cost-effective preparation methods, photocatalytic activity, and magnetic properties, etc. [4,5]. Researchers have paid more attention to ZnO semiconductors because of their high chemical stability, a high electrochemical coupling coefficient, a large exciton binding energy of approximately 60 meV, a vast energy bandgap of about 3.37 eV, a low dielectric constant, and good thermal conductivity, antibacterial, and UV-protection [6].

Furthermore, ZnO films are supposed to be excellent electroconductive and translucent piezoelectric materials that range from the infrared (IR) to the visible spectrum with significant refraction coefficients. As a result, ZnO could be used for ultraviolet absorbers, emitters, sensors, solar cell windows, antibacterial agents, and varistors. In addition, ZnO semiconductors have been recognized to be attractive photocatalysts due to the significant efficiency of catalytic, environmental sustainability, and reasonable price [7]. ZnO nanomaterials have been developed as effective catalysts for water detoxification due to the efficient generation of H_2_O_2_ and high mineralization and reaction rates. ZnO also has larger dynamic positions with large surface reactivity [8]. ZnO was one of the best photocatalysts for wastewater treatment because of its ability to be photocatalytic with ultraviolet (UV) light. The photoinduced recombination among the hole and electron, on the other hand, makes ZnO less useful in industrial applications [9].

It was mentioned before that the ZnO photocatalytic degradation efficiency enhances to certain organic compounds [10,11]. Similarly, pure and doped ZnO semiconductors are frequently utilized in wastewater treatment due to the dyes’ degradation of the paper printing, textile, wood, rubber, plastics, and silk industries [12]. Moreover, organic colorants, including Rhodamine B, Congo Red, Reactive Red 120, Methylene Blue, Methyl Orange, and Eriochrome Black T, are cancer-causing and poisonous, resulting in severe animal and human health effects [12]. Based on the current literature, enhancing photocatalytic activity has been intensively investigated by several researchers. Various methods have increased the photocatalytic activity of ZnO materials by adjusting the ZnO nanoparticles’ shape and size [13].

Various techniques of synthesizing have been inspected, including sol–gel [14], solid-state reaction, chemical precipitation, microwave radiation, solvothermal, and hydrothermal [15], to enhance the ZnO photocatalytic behavior. In addition, metal or non-metal dopants have improved ZnO optical, chemical, and physical properties. Sulfur dopants, for example, can raise the ZnO lattice constants and the oxygen vacancy. Likewise, a gold dopant might minimize the ZnO bandgap, while chromium dopants could increase the absorption of visible light [16].

Additionally, many scientific studies have confirmed that doping ZnO nanoparticles has positively improved ZnO’s physical characteristics, such as ferromagnetic conductivity, transparency, and shortened work function [17]. Furthermore, various studies have reported the effectiveness of doping NPs with either n-type or p-type semiconductor elements, or metals [18,19,20]. Lately, rare-earth elements have been utilized as dopant fillers to enhance the luminescence properties of semiconductors. Several rare-earth elements, such as erbium (Er-), europium (Eu-), lanthanum (La-), and dysprosium (Dy-), have been successfully reported as dopants to upgrade the chemical and physical properties of zinc oxide, the host matrix [21,22].

Additionally, ZnO doped with rare-earth elements showed various outstanding optical properties. Among all rare-earth elements, lanthanum (La)-doped ZnO nanostructures display exceptional structural, optical, electrical, gas sensitivity, and photocatalytic activity properties for different applications. Lanthanum (La) is a somewhat silvery-white soft metal in its pure form with an atomic weight of 138.9055 and an atomic number of 57, and it quickly tarnishes in the air [23]. The ionic +3 oxidation is the primary goal of the La-interaction. A naturally occurring element, lanthanum, can be added to steel in small amounts to increase its malignancy [23]. Up to the current research knowledge, no sufficient studies concerning the rare-earth effects on the structural, optical, electrical, energy bandgaps, and photocatalysis properties of the ZnO composite nanomaterials, mostly due to the difficult growth of doping procedures.

The present study investigated the structural, optical, electrical, and photocatalytic characteristics of various concentrations of La_2_O_3_-doped ZnO nanostructured composites. The combustion method of low cost, high quality, simple processing, low temperature, and effectiveness was applied to synthesize the proposed materials. The main goal is to create La_2_O_3_-ZnO nanostructures that can be used in numerous environmental, technological, electronic (as varistors), and photocatalysis applications. The studied samples will be further investigated for their structural, morphological, optical, and electrical varistors application through X-ray diffraction (XRD), scanning electron microscopy (SEM), Fourier transformation infrared spectroscopy (FT-IR), UV-Vis diffuse reflectance spectroscopy, current–voltage measurements, and dielectric analysis. In addition, RhB and MB were utilized to evaluate undoped ZnO and La_2_O_3_-ZnO nanostructured photocatalytic performance via visible photodegradation examinations.

## 2. Materials and Methods

### 2.1. Material Growth

A low-cost combustion process was applied to synthesize ZnO nanoparticles (NPs) doped with different concentrations of La_2_O_3_ rare-earth element. In the beginning, five grams of the Zn(NO_3_)_2_·6H_2_O mixture were mixed and ground in ceramic crucibles with one gram of gum acacia. The essential step is to add eight concentrations of lanthanum ions from 0.001 g to 5 g to the prior combination, where they are dissolved in 5 mL distilled water, which was named (S0–S7) as shown in Table 1. Next, the mixture of the produced La_2_O_3_-ZnO nanostructure composites was calcined for 2 h to 600 °C and then left at room temperature to cool down, after which they were ready to be used. The benefit of using gum acacia as fuel was that the ZnO structure transition, from crystallinity to the nanoscale, is supported by widening its constituents inside the host matrix. Finally, the process of a handheld hydraulic press was employed to form a pellet of the prepared La_2_O_3_-doped ZnO composite with 1 mm thickness and 13 mm diameter.

### 2.2. Instruments

The XRD is a vital tool for examining the structural phases of the prepared nanostructures using the Shimadzu LabX-XRD-6000 instrument with *CuK_α_* (*λ* = 1.5406 Ǻ, *2θ =* 5° to 80°). A scanning electron microscope (SEM) of JSM-6360 and n operating voltage of 20 kV is used for morphology studies. Furthermore, the UV-Vis optical spectra were measured using Shimadzu, Japan, at 200 to 1600 nm. A THERMO SCIENTIFIC, Fourier-transform infrared spectroscopy (FT-IR) was used from 400 to 4000 cm^−1^. Moreover, the UV-Visible diffused reflectance spectra of the synthesized La_2_O_3_-ZnO nanostructures were verified through an integrated sphere device coupled to a 3600 UV/Vis/NIR spectrophotometer (Shimadzu, Japan). Here the light wavelength is between 200 nm and 1600 nm with a scan step of 5 nm, and the reference material was barium sulfate.

Theoretical/dielectric characteristics of the La_2_O_3_-ZnO nanostructures were determined using a computerized digital Keithley 4200-SCS with a frequency range of 3 kHz to 10 MHz at an ambient temperature of 25 °C. For the electrical/dielectric characteristics, a specifically built holder was used. Current–voltage and current–temperature are controlled by a unique PID circuit. The electrical circuit consists of a pico, digital ammeter (Model DPM-111/SVS labs Inc., Saratoga, CA, USA), a high-voltage power supply (Model EHT-11/SVS laboratories Inc., Saratoga, CA, USA), and a PID-controlled oven (type PID–200). The operational system is linked to a separate oven equipped with a holder of two probes. Zn ranged with different concentrations of La_2_O_3_. In this current study, ZnO doped with varying concentrations of La ions was crushed in 10 tons on a solid disc with a thickness of 1 mm and a diameter of 13 mm, the photoreactor was furnished with a multi-position magnetic stirrer (i.e.,15-position installed in a multi-position device) under several experimental requirements. A wooden photoreactor under visible light irradiation was applied to test the considered samples for industrial wastewater treatment under different experimental conditions. The photocatalytic degradation process was studied in a photochemical reactor (eight lamps of 18 W, 60 cm, and a wavelength larger than 420 nm) [24].

For photodegradation, Rhodamine B (RhB) and Methylene Blue (MB) (10 mg/L) were investigated. In 200 mL of each aqueous solution, 0.1 g of ZnO or La_2_O_3_-ZnO nanocomposites were dispersed. After 40 min of stirring in the dark, the prepared mixture reached adsorption equilibrium. After that, it was exposed to light in a photochemical reactor. Before measuring Rhodamine B, and Methylene Blue concentrations, 2 mL of the reaction mixture was removed and centrifugated every 10 min to obtain photocatalyst particles. The concentration of Methylene Blue (MB) and Rhodamine B (RhB) in the solution has been analyzed via the following absorbance at 510and 665 nm, respectively, using a JASCO, UV-Vis-NIR-V-57 dual-beam spectrometer.

## 3. Results and Discussions

### 3.1. Structural Studies

#### 3.1.1. XRD Studies

**Figure 1:**
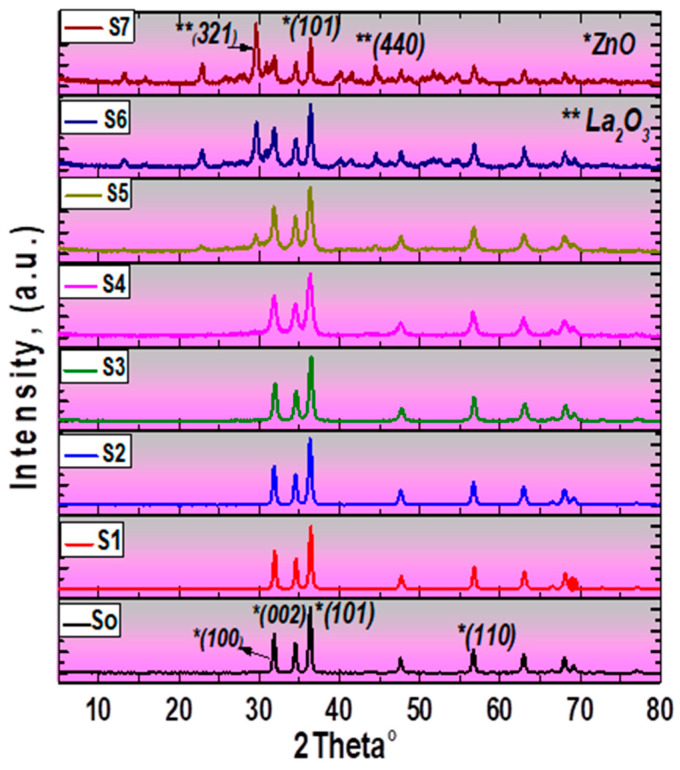
XRD patterns La_2_O_3_-ZnO nanostructured composites (S1–S7).

Figure 1 illustrates the XRD patterns of pure ZnO and La_2_O_3_-doped ZnO nanostructures. The figure clearly shows that the proposed composites demonstrate a wurtzite phase confirmed by JCDPS card No. 01-075-0576 with a carbon phase with related weak lines according to JCDPS card No. 01-074-2329 [12]. From the XRD diffractogram, the appeared La_2_O_3_-ZnO phase in the prepared composites was matched according to JCDPS card No. 03-065-3185. The prominent typical orientation peaks located at 31.7°, 34.5°, 36.3°, and 56.7° diffraction angles, respectively, corresponding to (100), (002), (101), and (110) planes were detected in the investigated La2O3-doped ZnO composite materials.

For the undoped ZnO material, especially for the (002) plane, a slight shift towards value to the left of theta was undoubtedly examined because of this incorporation of the RE-metals into ZnO nanoparticles [12]. Moreover, the main typical diffraction peaks located at 29.1° and 45.3° appeared with the highest ratio of lanthanum oxide doping in the host ZnO matrix (S6, S7), which were associated with the Miller indices of (321) and (440), respectively. Those XRD peaks indicated a hexagonal structure of the prepared La_2_O_3_-doped ZnO materials. These XRD results matched the reported structural data of sol–gel prepared RE-metals/ZnO nanocomposites by U. Alam et al. [12]. The XRD data was analyzed using Scherrer’s equation, and the crystallite size (*D*) of the synthesized La_2_O_3_–doped ZnO nanocomposites was determined as follows [25]:

(1) D=0.9λ/βcosθ,

Each synthesized La_2_O_3_-doped ZnO nanocomposites had a mean crystallite size varying from 13 nm to 40 nm. The grain size of the La_2_O_3_-doped ZnO nanoparticles is similar to the results of D.Venkatesan et al. [26]. Furthermore, the dislocation density (η) and the lattice strain (ε) of the ZnO matrix and La_2_O_3_-ZnO nanocomposites were evaluated through the following expressions [27]:

(2) $$\eta \;=\;1\;/D^{2},$$

**Figure 2:**
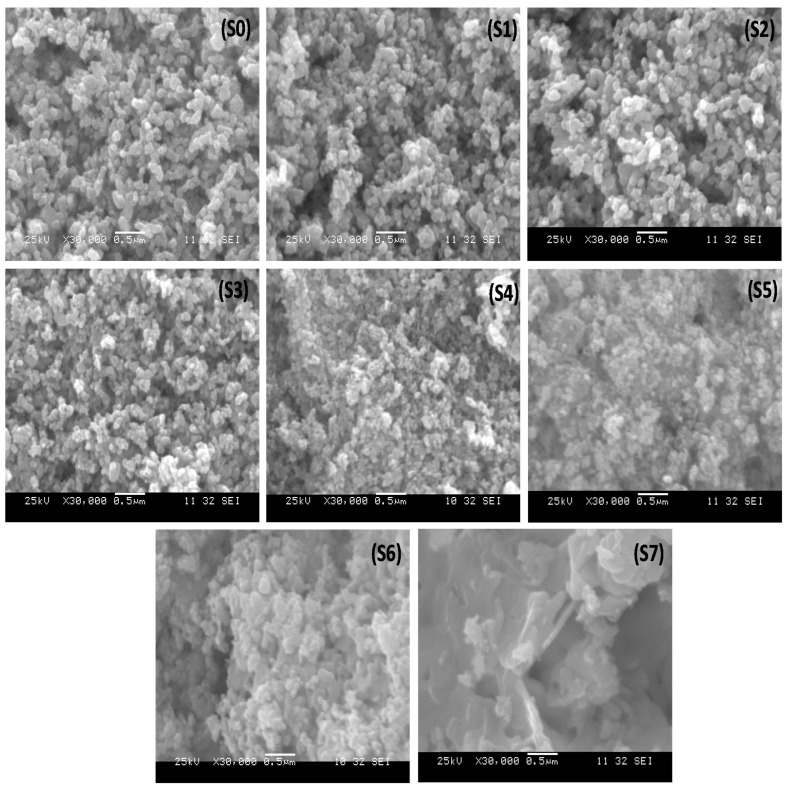
SEM pictures of the synthesized La_2_O_3_-ZnO nanocomposites at various concentrations.

(3) ε=βcosθ/4,

Here, *λ* is the X-ray wavelength (in nanometers), *θ* is the angle at which it diffracts in degree, and *β* is the full width at half-maximum (FWHM) in radians. Analysis of XRD data reveals that lanthanum significantly affects ZnO nanopowders’ crystalline structure. The calculated structural parameters of all the synthesized La_2_O_3_-ZnO nano-samples are shown in Table 2. The La_2_O_3_-ZnO-phase does not appear at small doping levels but at higher ratios (Table 2 and Figure 2). Similar results were conveyed by S. Anandan et al. They found that particle size reduces as the dopant ratio increases [28].

#### 3.1.2. SEM Analysis

The growth morphology of as-prepared La_2_O_3_-ZnO nanocomposites with different dopant ratios was investigated using the scanning electron microscope (SEM). Figure 2 shows SEM images of the La_2_O_3_-ZnO nanocomposites (S1 to S7). Undoped ZnO and lanthanum oxide (La_2_O_3_)-ZnO nanocomposites had markedly different structural morphologies. The La_2_O_3_-ZnO nanocomposite SEM pictures indicated a homogeneous dispersion of spherical La_2_O_3_ nanoparticles. Lanthanum ions increase crystal nucleation by forming tiny grains, but lanthanum grain entrapment hinders grain formation. This could be due to the ionic radius mismatch between zinc and lanthanum [29]. Alam et al. have reported the structural morphology of the Nd-doped ZnO nanocomposites and indicated that pure ZnO exhibits spherical particles of varying sizes. Nd-doped samples show a rough surface of a 15 nm average size [12]. The obtained SEM results agree with the reported data suggesting that La_2_O_3_ considerably affects pure ZnO.

### 3.2. Optical Studies

#### 3.2.1. FT-IR Spectroscopy

**Figure 3:**
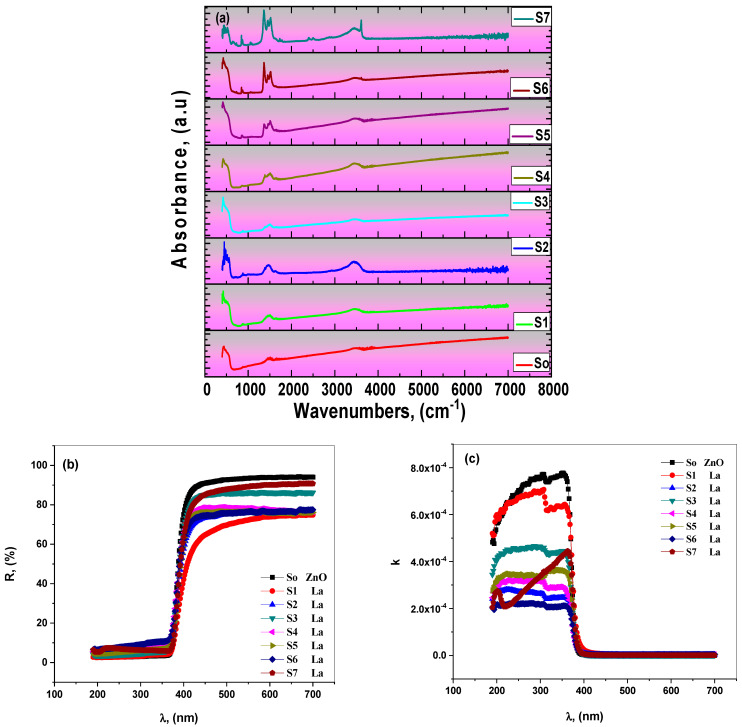
(**a**–**c**): (**a**) FT-IR spectra, (**b**) diffused reflectance (ODR), and (**c**) absorption index (k) of the as-prepared La_2_O_3_-ZnO nanostructured composites at different lanthanum oxide concentrations (S1–S7).

The vibrational properties of the La_2_O_3_-ZnO nanocomposites were investigated using FT-IR spectroscopy. FT-IR is a powerful investigative tool that may support XRD results. Figure 3a shows the FT-IR spectra of the prepared samples in the wavenumber raged from 400 to 7000 cm^−1^. It was observed from the figure that lanthanum oxide influences the intensity and the shape of the prominent absorption bands more than the undoped ZnO sample. There is a clear overlap of IR lines in the FT-IR spectral range, contributing to the ZnO and La_2_O_3_ phases. The optical transmission regarding the undoped ZnO sample was slightly improved for the low lanthanum concentrations. The transparency of La_2_O_3_-ZnO nanocomposites decreased with lanthanum concentration, possibly because of the increased scattering. The highest doping ratio of La_2_O_3_ (i.e., S7) has the maximum absorbance, corresponding to obtained results from both XRD and SEM investigation. Sowri Babun et al. investigated the FT-IR effects of ZnO/mesoporous silica nanocomposite, where the wideband located at about 3450 cm^−1^ relates to the stretch in OH vibrations [30]. They also observed a discrete peak at about 1630 cm^−1^ corresponding to the bending phases in the adsorbed water. Compared to the FT-IR spectra of metal nanoparticles (MNP), the MPS-doped ZnO nanostructured composites peak at 962 cm^−1^, where the obtained FT-IR results indicated the combination between ZnO and MPS.

#### 3.2.2. Diffused Reflectance Analysis

The measurement of diffuse optical reflectance (ODR) is a standard method for gathering information on the absorption characteristics of nanomaterials. Optical bandgaps and absorption index for semiconductors are two metrics from ODR analysis. The ODR and absorption index (k) of the prepared La_2_O_3_-ZnO nanostructured samples are shown in (Figure 3b,c) Figure 3b illustrates the ODR spectra of all examined La_2_O_3_-ZnO nanostructures in the 200–370 nm range. Within the ODR, a dramatic rise from 370 nm wavelength to 410 nm was seen, supplying the optical energy bandgap. The ODR results showed essential curves with no variations at 410–700 nm [30,31]. Alam et al. studied the sol-gel ZnO nanoparticles doped with various rare-earth metals of La, Nd, Sm, and Dy. The doping effects on the optical characteristics of ZnO were evaluated [12].

UV-Vis DRS analysis was used to explore the impact of RE-metal doping on the ZnO optical properties. From absorption data of undoped ZnO, an edge at around 381 nm is attributed to electronic transitions between the valance and conduction states. Alternatively, the absorption spectra of all REEs-doped ZnO nanocomposites illustrated a shift in the direction of longer wavelengths, indicating the effect of RE metals on ZnO nanoparticles [12]. As a result of the surface area of non-absorbent, absorption index values (k) of La_2_O_3_-ZnO nanocomposites are depicted in Figure 3c. The obtained data illustrated that the maximum absorption at around 380 nm is due to electronic band transitions’ characteristics (*π*→π*) [32,33]. Aydın et al. calculated the absorption coefficients of ZnO doped with iron metals. They found the k value was minimal, around 10^−4^ [31,32]. Those low absorption coefficient values are compatible with the proposed La_2_O_3_-ZnO nano-samples calculated optical parameters, concluding the doping effect. 

Tauc’s formula was applied to determine the optical energy bandgaps (*E_g_*) of the produced La_2_O_3_-ZnO nanostructured samples [34,35]:

(4) $$F(R)=\frac{{{}^{\left(1-R\right)}}^{2}}{2R}=\frac{K}{S},$$

(5) $$\alpha =\frac{F(R)}{t},$$

(6) $${\left(\alpha h\nu \right)}^{1/n}=A^{1/n}(h\nu -E_{g}),$$

where *F*(*R*) is the material reflectivity. *R* stands for diffuse reflectance (DR), *K* stands for the molar absorption coefficient, and *S* stands for the scattering amount. From Equations (5) and (6), *α* is called the absorption index, *t* is thickness, h is the photon energy, *υ* is the photon frequency, h is Planck’s constant, and A is the band tailing factor, having values between 1 × 10^5^ and 1 × 10^6^ cm^−1^·eV^−1^ [36]. As a result, the material direct bandgap and indirect bandgap are calculated using the following expressions [34]:

(7) $${\left(\alpha h\nu \right)}^{2}=A^{2}(h\nu -E_{g}),\;(n=½\;\mathrm{for}\;\mathrm{direct}\;\mathrm{allowed}\;\mathrm{transition})$$

(8) $${\left(\alpha h\nu \right)}^{1/2}=A^{1/2}(h\nu -E_{g}),\;(n=2\;\mathrm{for}\;\mathrm{indirect}\;\mathrm{allowed}\;\mathrm{transition})$$

**Figure 4:**
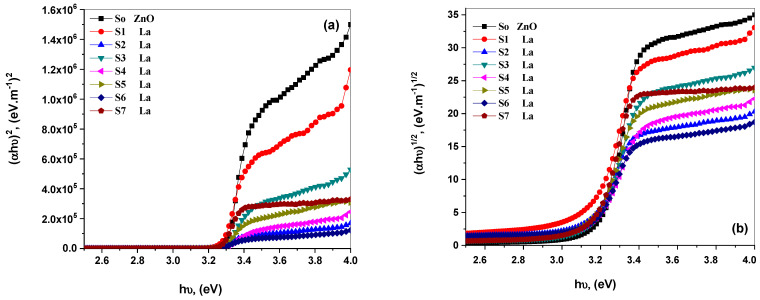
(**a**,**b**): (**a**) direct transition (*αhυ*)^2^, (**b**) indirect transition (*αhυ*)^1/2^ versus the photon energy (*hυ*) of the as-prepared La_2_O_3_-doped ZnO nanostructures.

Figure 4a,b plotted the (*αhυ*)^1/2^ and (*αhυ*)^2^ plots versus the photon energy ((*hυ*)) of prepared samples. The optical bandgaps were achieved separately by bringing the curves’ intercept line to the *x*-axis = 0 (i.e., the *x*-axis for *α*^1/2^ and *α*^2^ plots reach zero). For as-prepared La_2_O_3_-ZnO nanocomposites, the direct optical bandgaps are 3.32 eV to 3.24 eV, and indirect bandgaps are between 3.28 eV to 3.11 eV. Table 3 shows that as the dopant percentages increased, the optical energy bandgaps for samples decreased. These calculated values of present samples are well agreed with the reported data of Alam et al. [13]. His studies reported that the rare-earth-doped nanostructures show bandgap values of 3.25 eV to 3.17 eV [12]. Furthermore, T. H. AlAbdulaal and I. S. Yahia characterized the optical properties of TiO_2_/PMMA polymeric, where they concluded that the bandgap values decrease as the doping levels rise [37]. Therefore, in the present case, a similar trend of decreased bandgap suggests reducing circumstances point defects.

#### 3.2.3. Dielectric and AC Electrical Conductivity Studies

The dielectric function ε*(*ω*) is a direct response to electromagnetic radiation (EM) that will provide crucial information about the studied samples. The ε*(*ω*), *ε*_1_(*ω*), and *ε*_2_(*ω*) were calculated using the following equations [38]:

(9) $$\varepsilon^{*}(\omega )=\varepsilon_{1}(\omega )+i\varepsilon_{2}(\omega )$$

(10) $$\varepsilon_{1}\omega =\frac{C\times l}{\;\varepsilon_{0\;\;}\;\times A\;\;},$$

**Figure 5:**
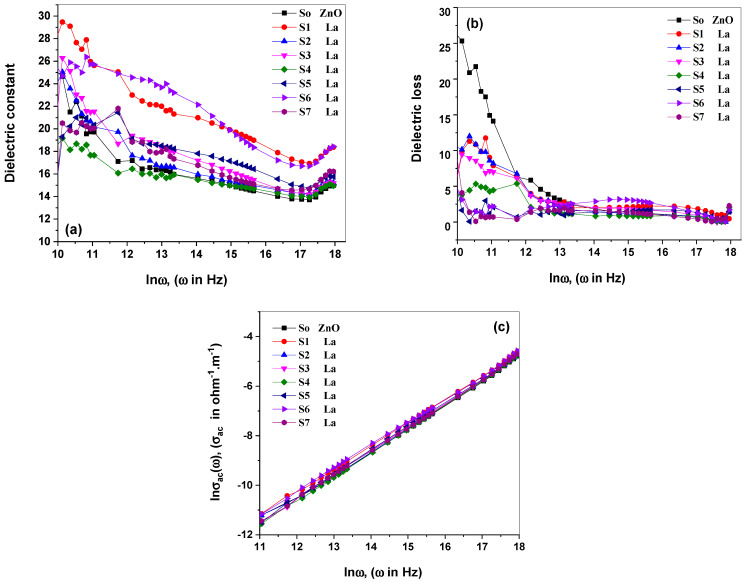
(**a**–**c**): (**a**) dielectric constant (**b**) dielectric loss (**c**) AC electrical conductivity (*hυ*) of the as-prepared La_2_O_3_-nanostructures.

*ε_2_(ω)* = tan δ × ε′, (11)

where the real part of the dielectric constant is *ε*_1_(*ω*), and the imaginary component is *ε*_2_(*ω*). A represents the electrode area, tanδ represents the loss tangent, *C*, *l*, and Z represents the sample capacitance, thickness, and impedance. The real dielectric constant ε_1_(ω) designates the light refraction. The imaginary part *ε*_2_(ω) correlates to the dielectric loss, primarily described as the electrons’ transition between the occupied and empty levels. Figure 5a,b illustrates the difference between *ε*_1_(*ω*) and *ε*_2_(*ω*), varying between 3 × 10^3^ Hz and 10^7^ Hz, respectively. Figure 5a shows that the dielectric constants decrease significantly in low-frequency regions. The photon frequency increases (as high as 16), then the dielectric constants decline marginally as the photon frequency rises.

The nanostructured La_2_O_3_-ZnO revealed a substantial polarization of the low-frequency region when the dielectric constants were improved with frequency reduction. When the frequency approached around 13, the dielectric constant values grew until they reached their maximum value (about 35). Dielectric constant values in La_2_O_3_-ZnO nanostructures fall faster with larger lanthanum doping ratios. Furthermore, the dielectric loss declines to the smallest possible value before increasing again. Aydın et al. illustrated that the dielectric parameters decrease as the photon frequency increases [34]. Dielectric polarization arises as ionic, electronic, dipolar, or interfacial polarization. In the high-frequency region, ionic and electronic mechanisms are dynamic, whereas the dipolar and interfacial polarizations are active in the low-frequency area [38]. In the low-frequency range, *ɛ*_1_(*ω*) and *ɛ*_2_(*ω*) increase could be accredited to the interfacial mechanism that happens through the space of the created charges in the material, which persuade image charges on electrodes. With an external electric field, the migrated space charges are trapped via energy defects, where a localized charge accumulates at the electrode and the interface of the material, increasing the dielectric parameters in low-frequency regions [37]. At high frequency, the axis *ε*_1_(*ω*) starts to grow, taking advantage of the oscillation of the dipole, which could rotate rapidly [39].

The following formulae were used to determine the AC conductivity as follows: [40,41,42,43]:

(12) $$\sigma_{Total\;.AC}\left(\omega \right)=\frac{t}{ZA},$$

(13) $$\sigma_{Total\;.AC}\left(\omega \right)=\sigma_{DC}\left(\omega \to 0\right)+\sigma_{AC}\left(\omega \right),$$

(14) $$\;\sigma_{AC}\left(\omega \right)=B\omega^{S},$$

The total AC electrical conductivity is $\sigma_{Total.\;A.C}.\;\left(\omega \right)$, *Z* is the impedance, and *A* is the effective cross-section area. The *B* is a temperature-dependent constant describing the conductivity diffusion of the studied medium. Furthermore, $\sigma_{DC}\left(\omega \to 0\right)$, $\sigma_{AC}\left(\omega \right)$ for DC and AC electrical conductivities. Where *ω* is the angular frequency and *s* is the frequency exponent constant. In Figure 5c, the computed values of *lnσ_AC_*(*ω*) were compared to *ln*(*ω*) for all generated La_2_O_3_-ZnO nano-samples. The electrical conductivity of the produced La_2_O_3_-ZnO nanostructures increases linearly with the incoming frequency and the concentration of the lanthanum/ZnO matrix. The frequency exponent (*s*) was estimated at room temperature from the slope of the obtained results in Figure 5c. The determined s values in this investigation were estimated to be equal (1) [40,44]. The AC electrical conductivity commonly depends on the applied frequency for disordered, ordered, and nanomaterials [45]. The semiconductor materials displayed AC electrical conductivity values between 10 × 10^−9^ and 10 × 10^3^ Ω^−1^·cm^−1^ [45].

The frequency exponent (*s*) value, which ranges from zero to one, is determined by the frequency and the temperature. On the other hand, the traditional Debye-type medium is equal to one. Impurities are created by inessential electrical dipoles or charge carriers connected to the frequency parameter (*s*). The frequency exponent values ranged from 0.6 to 0.8 for disordered mediums, about 1 in the case of dielectric mediums with highly disordered mediums [46,47]. Aydın et al. studied Fe-doped ZnO nanocrystalline materials and concluded that electrical conductivity declined as the dopant ratio increased [34]. The activation energy differed from various conduction mechanisms related to the defect levels among the optical energy bandgaps. Intrinsically, the defect of energy levels was generated, and the defect sites in the optical bandgaps varied as a function of dopants [34].

### 3.3. Electrical Properties

Pre-breakdown, breakdown, and upturn areas dominate the V-I characteristics [48]. The current (*I*) has a high nonlinear relation with the applied voltage (*V*), which could be calculated through the empirical law [49]:

(15) $$I=KV^{\alpha },$$

**Figure 6:**
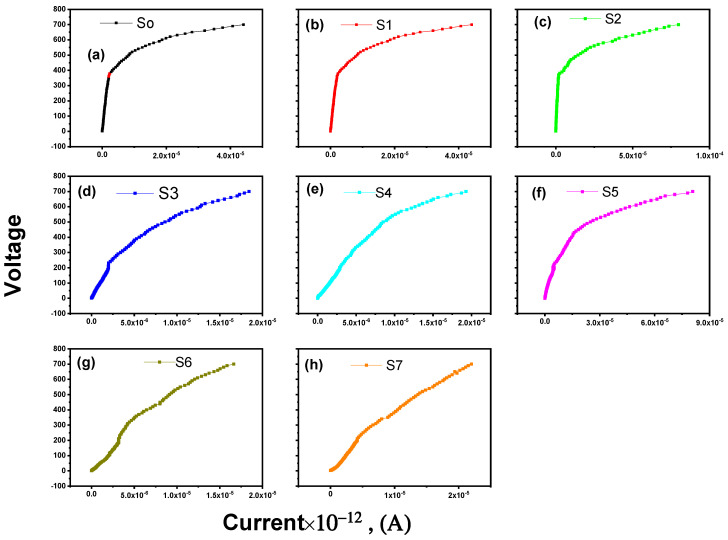
(**a**-**h**): V-I characteristics for the as-prepared La_2_O_3_-ZnO nanostructures.

*K* is a constant that varies depending on the manufacturing and geometry procedure. The slope of the inverse of the *lnV-lnI* plots at any applied voltage value computes the nonlinear coefficient. Figure 6a–h illustrates the associations among the applied voltage (*V*) and the current (*I*) of the undoped ZnO and La_2_O_3_-ZnO nanostructures with various lanthanum ratios. Based on ceramic varistors, all the prepared samples illustrate apparent nonlinear performance. The obtained outcomes are remarkable comparisons with the conclusions by Xu et al. [50]. The conduction properties can be split into two regions: a linear region with a high impedance lower than the voltage of the knee-point and a nonlinear part with low impedance over the knee-point. The behavior of all specimens is markedly nonohmic. According to the curves, the conduction properties are separated into a high-impedance linear region below the knee-point voltage and a low-impedance nonlinear region above the knee-point voltage.

Nahm et al. observed that the knee-point voltage was sharper among the two zones in all plots that described the improved nonlinear behavior [51]. The exact nonlinear property can expose the ceramic varistor specimens’ nonlinear absorption coefficient (*a)*, leakage current, and threshold voltage (*V_ss_*). The AC impedance parameters analyzed could be utilized to investigate the complex impedance (Z* = Z′ *+ iZ*″) using the following functions below [52]:

(16) $$Z^{\prime }=\frac{1}{\omega c_{o}}\left[\frac{\varepsilon 2\left(\omega \right)}{\varepsilon 1{\left(\omega \right)}^{2}+\varepsilon 2{\left(\omega \right)}^{2}}\right];\;Z^{\prime \prime }=\frac{1}{\omega c_{o}}\left[\frac{\varepsilon 1\left(\omega \right)}{(\varepsilon 1{\left(\omega \right)}^{2}+\varepsilon 2{\left(\omega \right)}^{2}}\right],\;$$

Here, ω is the chosen angular frequency, the real and imaginary sections of the dielectric permittivity are *ε*_1_(*ω*), *ε*_2_(*ω*) *=* *ε*_1_(*ω*) *tanδ* and the vacuum capacitance is *c_o_*. The impedance components were determined using the following expressions [52]:

(17) $$Z^{\prime }=\frac{R_{gb}^{2}Q\omega^{c}\cos\left(\frac{c\pi }{2}\right)+R_{gb}}{{\left(1+R_{gb}Q\omega^{c}cos\left(\frac{c\pi }{2}\right)\right)}^{2}+{\left(R_{gb}Q\omega^{c}sin\left(\frac{c\pi }{2}\right)\right)}^{2}},$$

(18) $$-Z^{\prime \prime }=\frac{R_{gb}^{2}Q\omega^{c}\sin\left(\frac{c\pi }{2}\right)}{{\left(1+R_{gb}Q\omega^{c}cos\left(\frac{c\pi }{2}\right)\right)}^{2}+{\left(R_{gb}Q\omega^{c}sin\left(\frac{c\pi }{2}\right)\right)}^{2}},$$

**Figure 7:**
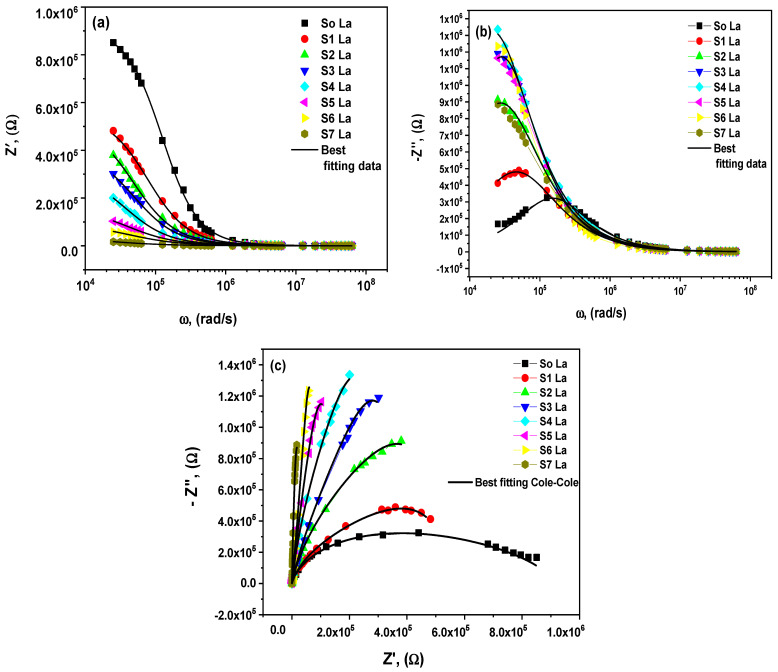
(**a**–**c**): (**a**) real, (**b**) imaginary parts, and (**c**) Cole-Cole plots (the line represents the theoretical best Cole-Cole fitting) as-prepared La_2_O_3_-ZnO nanostructures.

The average square method was used for simulating the resistance (*R_b_*). The difference between the experimental and theoretical results of the *Q* and *c* parameters indicates the value of the CPE impedance as *Z_CPE_ =* 1/*A*_0_(*iω*)*^c^*. The resistance of grain boundary (GB) was determined from the Cole–Cole plots of the impedance data. It uses Equations (17) and (18), where Figure 7a,b presents the $Z^{\prime }\left(\omega \right)$ and $-Z^{\prime \prime }\left(\omega \right).$ The undoped ZnO and La-doped ZnO nanostructures with various lanthanum contents at room temperature. Using Equation (17), this perfectly matches the fundamental part of the AC impedance’s experimental results. Because of the resistive grain boundaries’ efficiency in the region of lower frequency, the most significant $Z^{\prime }\left(\omega \right)$ values were observed that correspond to large resistivities [53,54]. This behavior could be described as the AC conductivity increasing as the angular frequency increases [54].

**Figure 8:**
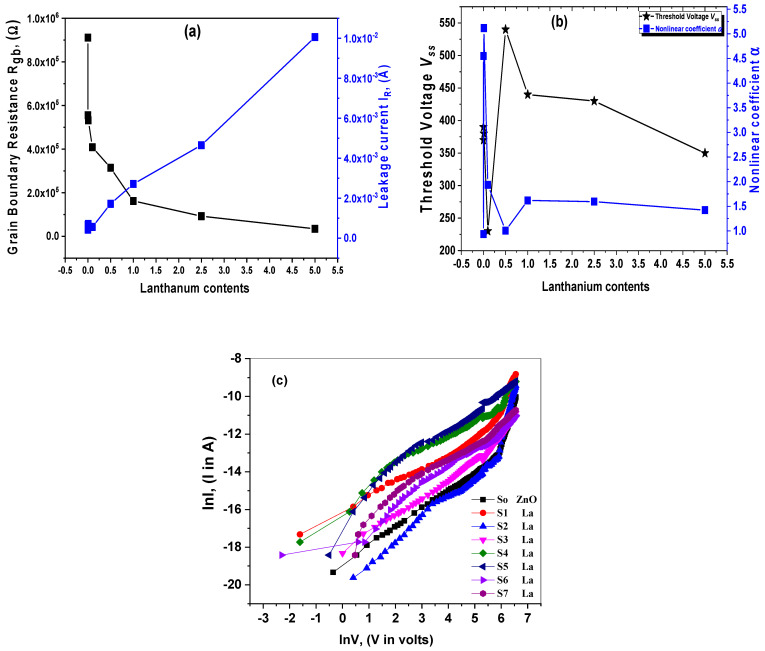
(**a**–**c**): (**a**,**b**) The V–I fitted as a function of the lanthanum contents, (**c**) linear relation between *lnI* and *lnV* as-prepared La_2_O_3_-ZnO nanostructures.

The angular frequency dependence of $-Z^{\prime \prime }\left(\omega \right)$ is shown in Figure 7b. The experimental results from Figure 8b closely match the theoretical data from Equation (18). Each of the $-Z^{\prime \prime }\left(\omega \right)$ curves exhibit a beginning peek of the relaxation process, which changes its intensity because of lanthanum doping. This indicates a decrease in the loss of dopant nanoparticles, as reported by A. Azam et al. [55]. Figure 7c demonstrates the Cole–Cole diagrams to obtain the specimens’ dielectric parameters as a function of the various lanthanum contents. Considering the studied corresponding circuit model nanomaterial for varistor applications is essential. This figure matches the fitting data and the practical results, indicating that the corresponding future circuit is appropriate for the material’s electrical properties. The obtained grain boundary of the circuit element value is displayed in Figure 8a. A comparable RC circuit was used to fit each semicircle, such as a parallel grouping of bulk grain boundary resistance $\left(R_{gb}\right)$ and bulk grain boundary capacitance (CPE). The undoped ZnO material’s bulk resistance is larger than the bulk grain boundary resistance. Correspondingly, it was seen that the grain boundary resistance $(R_{gb}$) declined due to lanthanum doping, where this concluded result agrees very well with the published data by Chedia Belkhaoui et al. [56]. It was noticed that with the rise of lanthanum content, the causes of the sharp increase in the leakage current, as shown in Figure 8a, are the breakdown of the Schottky barrier with inverse biased. The attained results were matched with the reported study by S.-G. Yoon et al. [57]. A graphical explanation of correlation plots between the lanthanum dopants, the nonlinear coefficient (*α*), and the threshold voltage (*V_ss_*) of the ZnO varistor nanocomposites is schematically summarized in Figure 8b. The optimum nonlinear coefficient α is assumed at (S2) La- content based on the obtained results. After adding La- content, the nonlinear coefficient can be minimized. When the lanthanum quantity increases to (S2) La, the material develops extremely resistive without any nonlinearity signs. This indicates that increasing lanthanum concentrations would not affect the nonlinear characteristics of current voltage (Figure 8c). The studied varistor nanocomposite threshold voltage and nonlinear coefficient vary adversely and irregularly with the increased La-doped content.

### 3.4. Photocatalytic Performance

The photocatalytic studies were performed using the photodegradation of MB and RhB for the proposed La_2_O_3_-ZnO nanostructured samples, where all photocatalytic data was operated under visible light. However, the obtained experiments were maintained in the dark until equilibrium before the light irradiation to study the adsorption process. At the same time, the MB and RhB adsorption over La_2_O_3_-ZnO was ignored.

**Figure 9:**
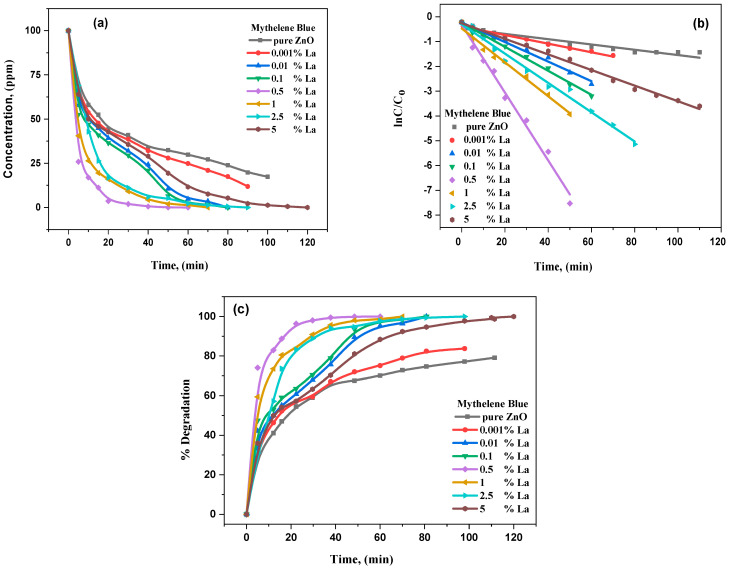
(**a**–**c**): photodegradation of MB in the presence of pure ZnO and La_2_O_3_-ZnO nanostructured composites, (**a**) concentration of MB with time, (**b**) kinetic study of photodegradation of MB, (**c**) % degradation of MB versus time.

**Figure 10:**
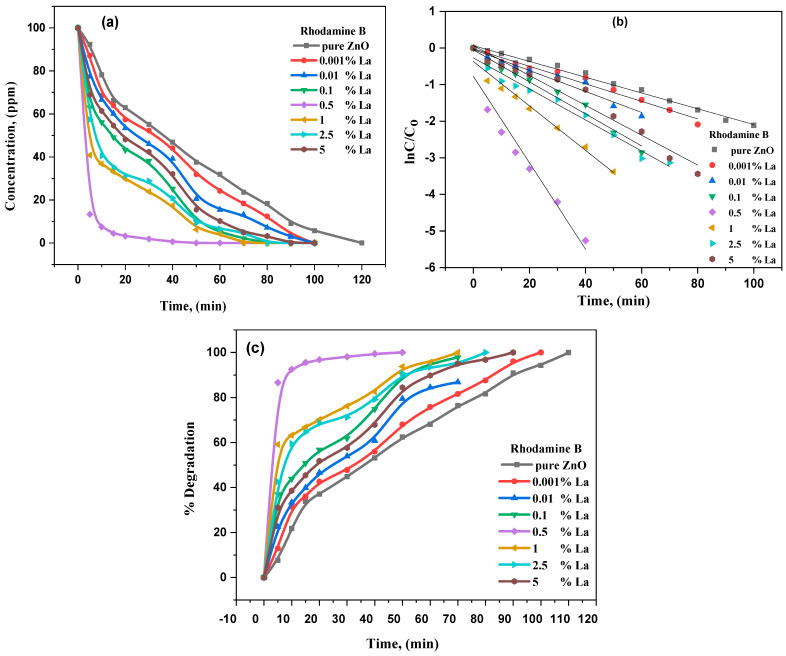
(**a**–**c**): photodegradation of RhB in the presence of pure ZnO and La_2_O_3_-ZnO nanostructured composites, (**a**) concentration of RhB with time, (**b**) kinetic study of photodegradation of RhB, (**c**) percentage degradation of RhB versus time.

Several experiments were conducted using MB and RhB in aqueous suspension under visible light to characterize the undoped ZnO and La_2_O_3_-ZnO nanostructures. The photocatalytic degradation tracks a pseudo reaction with the first order. The kinetics could be determined employing *ln(C_0_/C)* = *kt*, in which *k* is the constant reaction rate, *C*_o_ is the starting concentration of MB and RhB, and *C* is the MB and RhB concentrations at *t* of the reaction time. Figure 9 and Figure 10, respectively, illustrate the kinetic fit for the MB and RhB degradation on undoped and La-doped ZnO with different lanthanum concentrations. The linear relationship between the time and integral transform *ln(C_0_/C)* discloses the first order’s deceptive reaction kinetics.

Table 4 illustrates the values of the reaction rate constants (*k*) for La_2_O_3_-ZnO nanostructures. The degradation rate for the La_2_O_3_-ZnO photocatalytic is more significant than that for the undoped ZnO nanostructures. Interestingly, the catalyst’s rate constant increases to S4 concentration and decreases. The catalyst studies show that 0.5 g of La-doped ZnO nano-samples is more dynamic, which displayed the enormous photocatalytic performance for RhB and M.B. degradation. Therefore, the catalyst studies conclude that the ideal La-doping ratio is 0.5 g of La-contents, which could further help separate the photoinduced electron–hole pairs and improve photocatalytic degradation. The high photocatalytic effects of the La-doped ZnO nano-samples with 0.5 g of La^3+^ ions/ZnO could be because of the effective separation among the hole and electron pairs. The potential value should be higher than 0.2 V, as reported by Pleskov [58].

As the concentration of in La^3+^-ions grows, the barrier of La_2_O_3_-ZnO surface turns into larger values, and the potential region of space charges alters to be narrower. Subsequently, the hole and electron pairs are competently parted using the high electric field. In contrast, by increasing the La^3+^-ions’ concentration, the ZnO nanostructured light dispersion depth obtained could significantly surpass the space charge layer. Consequently, recombining the photogenerated pairs of electrons and holes becomes easier. The electrical field and the band-bending of ZnO’s colloidal particles are generally small, though the optical bandgaps of La_2_O_3_ rare-earth oxides are inadequate for photocatalytic effects. The ideal La^3+^-loading is essential for matching the charge layer’s thickness and the light penetration depth to separate the photogenerated pairs between hole and electron.

Additionally, the appropriate La^3+^-concentration is required to produce a significant potential variation among the particles’ center and surface to competently disperse the photoinduced pairs of electrons and holes [59]. The photocatalytic effects would be minor because the additional La_2_O_3_ casing the ZnO surface could raise the number of recombination centers. The photocatalytic results of La-doped ZnO nanostructures, with more than (S4) of La-dopants, are very low. The high photocatalytic activity of La-doped ZnO with (S4) La dopants is the strong O^.H.-^-ions absorption on the ZnO surface due to the oxygen vacancies. The surface-bound OH^--^ ions are expected to trap the photoproduced holes to avoid electron–hole recombination.

ZnO is well known to be a semiconductor photocatalyst in the photo-reductive dehalogenation for halogenated benzene derivatives, the photocatalytic reduction in the toxic ions of metals and the photocatalytic degradation of water pollutants. Commonly, most dyes are accepted to be remarkably resistant to direct photolysis and biodegradation. For instance, numerous N-containing dyes, Rhodamine B (RhB), experience expected degradation of reductive anaerobic to produce amines of carcinogenic aromatic [60]. Consequently, RhB dyes were preferred to be the perfect contamination to investigate the proposed nanostructures’ photocatalytic activity in this current research. The evaluation of photocatalytic performance for the RhB aqueous solution was completed at room temperature. Under dark conditions, photocatalytic measurements for undoped ZnO and La^3+^-ZnO nanostructures illustrated no difference with the RhB concentration. Simultaneously, the photocatalytic RhB decolorization was not accomplished using ultraviolet light for the proposed undoped and doped ZnO with La^3+^-composites. The results also concluded that the photocatalytic performance was enhanced by combining catalyst and light, directly degrading RhB decolorization. The distinctive absorption peaks are 664 and 553 nm for MB and RhB, respectively, and were utilized to track the photocatalytic degradation process. Figure 9 and Figure 10 represent the photodegradation of MB and RhB, correspondingly using undoped ZnO and La_2_O_3_- ZnO N.P.s under visible light conditions. The reactions follow the model of Langmuir–Hinshel. The kinetics, as well as the photocatalytic performance of the studied nanomaterials, the percentage degradation of MB and RhB can be determined by applying the following equation [60]:

*ln*(*C*/*C_o_*) → *kt*, (19)

*% of degradation* = (*C_o_* − *C*/*C_o_)* × 100%, (20)

where *C_o_* is known as the concentration at 0 min of irradiation time, and *C* is the concentration at t time with a constant rate of *k*. The concentration reduction in MB and RhB as a time function is represented in Figure 9a and Figure 10, respectively. The kinetic data of photodegradation of MB in Figure 9b demonstrated that the reaction rate constant varied between 0.013 and 0.130 min^−1^, ranging from 0.021 to 0.110 min^−1^. The degradation rate rises with the doping level till reaching a maximum at (S4) of La, decreasing it. The degradation efficiency of MB and RhB photodegradation versus time is illustrated in Figure 9c and Figure 10c, using the synthesized NPs. It was also ascertained that the photocatalytic activity rate is different from changing lanthanum concentrations in La_2_O_3_-ZnO nanomaterials. As the loading increased to (S4) of La^3+/^ZnO, the highest efficiency of photocatalytic activity was reached. To explain that, dissolving La^+3^-ions into the ZnO matrix would produce other surface defects. The obtained results were donated to enhance the photocatalytic activity of 0.5 g of La^3+^: ZnO nanomaterial, associated with the photocatalytic activity of both undoped ZnO and other La^3+^-doped ZnO nanostructures. Though an additional increase in La^3+^-dopants, such as 1 g or 2 g, of La^3+^-in ZnO, the matrix is expected to produce the La_2_O_3_ aggregation and other La–O–Zn chemical bonds, and the function of the formed region of surface charge is negatively affected, which was not successful in competently distinguishing the photogenerated pairs of electron–hole. Therefore, the photocatalytic performance of the 2 wt% of La^3+^: ZnO is very low compared to the (S5) La^3+^-doped ZnO nanostructures [61]. In the current work, the photocatalytic analysis concluded that the lanthanum dopant’s ideal concentration is (S4) of La^3+-^ions.

### 3.5. Scavenger Investigation and Photocatalytic Mechanism

ZnO semiconductors with large bandgaps have been considered a photocatalyst for the pollutants’ degradation in water or wastewater. UV irradiation is the main restriction of ZnO usage because of its sizeable optical bandgap. This ZnO limitation was overcome through lanthanum doping, which caused the ZnO bandgap to be narrower. Therefore, from visible light sources, La_2_O_3_-doped ZnO nanostructures can straightforwardly absorb photons. The electrons of the semiconductor were excited from the valance band (VB) to the conduction band (CB), which creates pairs of electrons and holes (e^−^ and h^+^), as illustrated in Equation (20). The VB holes could generate hydroxyl radicals (OH^●^) via the oxidative reactions as displayed in Equations (21)–(23), and the CB electrons could generate anions of superoxide radical (^●^O^2−^) via the reductive responses as illustrated in Equations (24)–(26). Both OH^●^ and ^●^O^2−^ are principal oxidants to oxidize organic compounds. The ^●^O^2−^ has adequate reduction capacities for oxidizing organic classes, which have electron-donating, solid groups, for example, -N=N- bond, active azo bond, etc., and OH^●^ inclines to bond C-C or abstract hydrogens unsaturated bonds [60]. Therefore, a model contaminant comprising unsaturated C-C bonds, such as MB and RhB, could be effortlessly attacked with OH^●^ or photo-catalytically oxidized. The MB and RhB degradations happened rapidly in the OH^●^ existence as follows:

(i) Light (appropriate spectrum) + La-doped ZnO → La-doped ZnO (h^+^ + e^−^), (21)

Oxidative reactions with holes h^+^ + H_2_O → H^+^ + OH^●^, (22)

2h^+^ + 2 H_2_O → 2 H^+^ + H_2_O_2_, (23)

H_2_O_2_ → OH^●^ + OH^●^, (24)

(ii) Reductive reaction with O_2_
2e^−^ + O_2_ → ^●^O^2−^, (25)

^●^O^2−^ + 2 H^+^ → H_2_O_2_ + O_2_, (26)

H_2_O_2_ → OH^●^ + OH^●^, (27)

**Figure 11:**
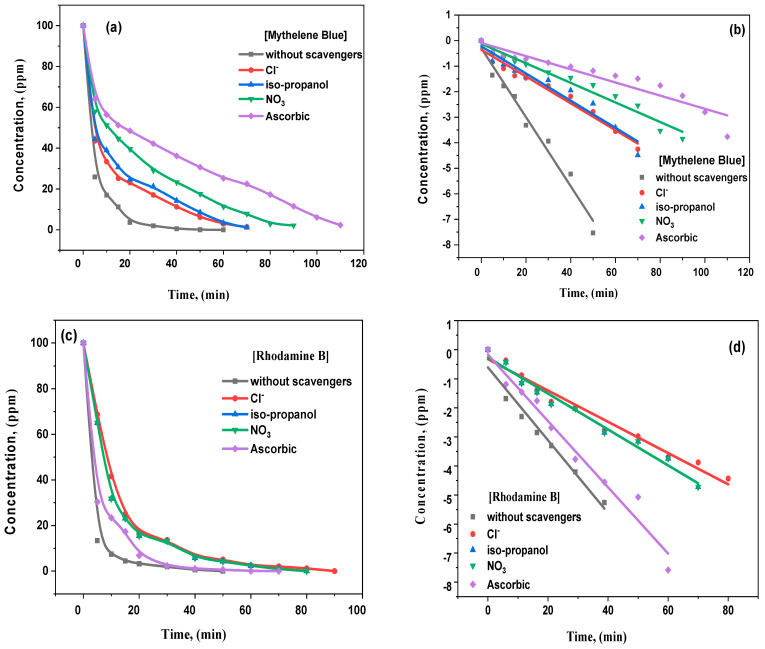
(**a**–**d**): Influence of different scavengers on the photocatalytic activity of pure ZnO and La_2_O_3_-ZnO nanostructured composites.

The scavenging agents explain the main reactive species, such as photo-created holes and electrons, OH^●^, or superoxide radicals, in RhB and MB molecules [61]. The scavenging measurement and analysis are shown in Figure 11. The degradation of 200 mM sodium chloride as a hole scavenger substantially diminishes the catalyst’s activity against MB photodegradation, as demonstrated in Figure 11a. The photogenerated holes could respond and produce chlorine radicals within the chloride anion. The chloride anion radical could quickly react and produce dichloride anion (Cl_2_) radicals, where two Cl_2_ Radicals can react straightforwardly to generate chlorine and chloride-free anions. Additionally, dichloride anion radicals can directly respond to water molecules to create chloro-hydroxyl (ClOH^−^) radicals, in which the reaction between Cl and H^+^ happens. Furthermore, chloride has been stated as the OH^●^ radicals’ scavenger for producing HClO**^−^**. As the primary varieties, the HClO^−^ and Cl results were at pH levels > 7.2 and <7.2. Thus, chloride anions in solutions make available the employed catalyst to achieve lower activity. Figure 11d illustrates the isopropanol impact (IPA) as a trapping agent.

Basic aliphatic diols and alcohols performed as trapping agents in scavenging OH^●^ radicals through the transformation into consistent ketones or aldehydes. The isopropanol absorbed OH^●^ radicals and produced radicals of (CH_3_)_2_ COH. The resulting radicals had less reactivity than OH^●^ radicals, dropping MB degradation at advanced IPA dosages [62]. The photo-created electrons’ response to water molecules produced hydroxyl and H ions. Moreover, IPA could scavenge at a relatively lower H rate than OH^●^ radicals. Comparing the obtained data for the IPA and Cl impacts confirms that Cl is exceptionally economical in decreasing MB degradation due to concurrently trapping h^+^ and OH^●^. As an electron confining occurs, sodium nitrate is realized in the findings of Figure 11. In nearly natural waters, slight concentrations of nitrite and nitrate ions are considered trapping agents. OH^●^ can trap the nitrite ions efficiently, even though the photogenerated electrons and H can be sifted to a lesser degree. The photogenerated electrons can effectively be scavenged through nitrate anions [63].

**Figure 12:**
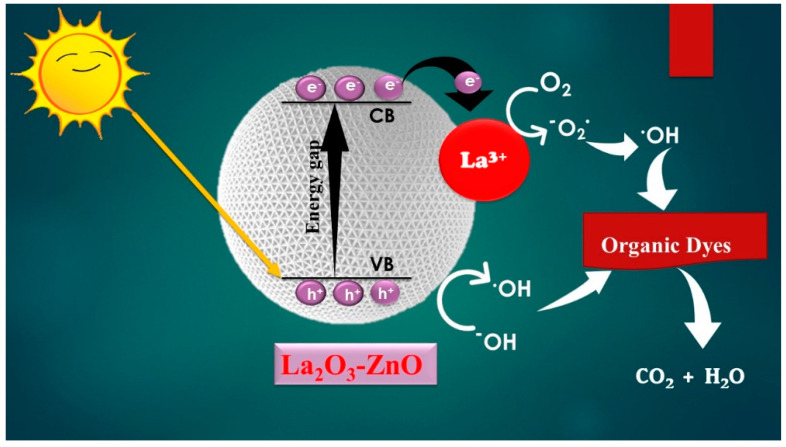
Photocatalytic mechanism of La_2_O_3_-ZnO nanostructured composites.

Ascorbic acid (H_2_A) demonstrated the most critical decrease in MB degradation, considering it an O_2_^−^ scavenger. In acidic conditions, H_2_A reacts with superoxide radicals, as well as a reaction of second order, producing H_2_O_2_ and ascorbate radicals. The ascorbate was relatively nonreactive and decayed into dehydroascorbic acid and H_2_A [64]. As approved previously, the subsequent model to degrade the composite’s photocatalytic activity was noticed in trapping agents, such as A.A.> IPA > Nitrate > Chloride. This model recommends the proposed nanocomposites in the MB photodegradation with superoxide radicals, and then OH^●^, e^−^, and h^+^ had critical positions. To conclude, semiconductors will be excited by the incident photons to produce electron and hole pairs. As a result, the photogenerated electrons will be allowed to transfer from the ZnO level’s conduction band (CB) to the CB of the La level. Similarly, the holes will move from the valance band (VB) of the La dopant to the VB of the host ZnO matrix because the VB-ZnO level has a higher negative potential compared to VB-La. These charge carriers’ transition mechanisms result in aggregating the photogenerated holes at the VB-ZnO level and the photogenerated electrons at the CB-La level, as represented in Figure 12.

### 3.6. Comparison of Photocatalytic Efficiency with Previous Work

To estimate the photocatalytic efficiency of the synthesized nanocomposites, a solution of MB and RhB was irradiated with ZnO and La: ZnO under visible light, as presented in Table 5. The photocatalytic efficiencies of (S4) of La_2_O_3_:ZnO nanostructures are more than the efficiencies of ZnO and other La-doped ZnO as-prepared and mentioned above, concluding the efficiency of this current prepared system. Interestingly, the obtained results agreed very well with the studies of mineralization and degradation. For comparison, the (S4) of La_2_O_3_: ZnO nanostructures has significant photocatalytic efficiency that was 2.5 times the ZnO efficiency. Furthermore, S. Anandan et al. found that the 0.8 g of La: ZnO nano-samples were the most powerful dynamic that displayed enormous photocatalytic performance and a significant relative photonic efficiency for the monocrotophos degradation [61]. S. Anandan et al. also obtained La: ZnO photocatalytic enrichment performance, primarily because of tiny La-particle size and the ability to reduce the recombination of pairs of electrons and holes [28]. Furthermore, the TCP adsorption increase over La:ZnO nanostructures could be associated with high photocatalytic activity.

Tikkun Jia et al. utilized Rhodamine B (RhB) ‘s degradation to investigate the photocatalytic performance of the undoped ZnO and La^3+^-ZnO nanostructured materials [65]. The attained results concluded that the concentrations of La^3+^-dopants extraordinarily affected the photocatalytic efficiency, where the ideal doping ratio was established to be 2 wt%. Sumetha Suwanboon et al. reported that the precipitation technique was more efficient than the mechanical milling technique in synthesizing La-doped ZnO nanostructures, which showed the maximum photocatalytic degradation of M.B. due to the oxygen vacancies [66]. Korak et al. established that 0.5 mol % of La: ZnO was the furthermost photocatalytic active with the metasystem degradation [67]. Finally, M. Shakir et al. stated that 3% of La: ZnO nanostructures displayed the most fantastic photocatalytic activity, confirmed through the 80% paracetamol (PARA) drug degradation in 180 min [68].

Lee prepared and applied La: ZnO photocatalytic materials for treating an aqueous solution of 100 mg/L paracetamol using visible irradiation after three hours [69]. The 1.0 wt% of La:ZnO nanostructure displayed the highest photocatalytic performance for paracetamol degradation with 9% efficiency [70]. R. Bomila studied the relationship between photocatalytic activity and La-dopants on ZnO, where the methylene blue degradation was investigated using solar irradiation [71]. Petronela Pascariu reached a maximum color removal efficiency of about 97.63% for ZnO nanostructure doped with 2% of La^3+^ as a rare-earth element in a 0.283 g/L dosage [72]. Finally, the improved catalyst of La:ZnO was thermally started at a temperature of 700 °C for one hour and then effectively recycled for methylene dye photodegradation. Loan T.T. Nguyen synthesized lanthanum (La)-doped zinc oxide nanoparticles and applied them to decompose methyl orange to achieve 85.86% within 150 min under visible light utilizing 0.1% of La with 0.9% of ZnO as the photocatalyst [73].

### 3.7. Recycling and Reusability Analysis

**Figure 13:**
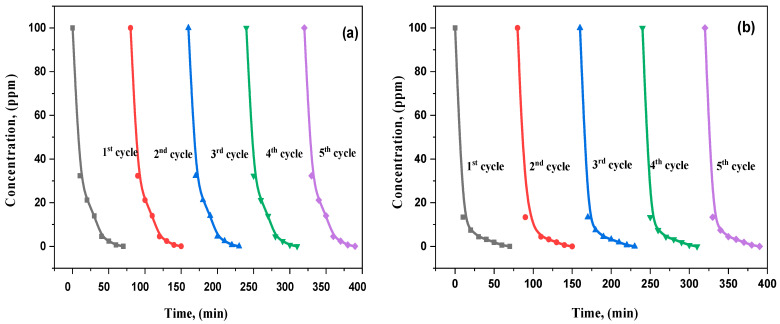
(**a**,**b**): recycling runs of (S4) La_2_O_3_-ZnO nanostructured composite for photodegradation of (**a**) MB, (**b**) RhB.

The durability and reusability of the La_2_O_3_-doped ZnO nanostructured nanocomposites were investigated through visible radiance as a characteristic test for the photodegradation of M.B. and RhB. Figure 13 displays the photocatalytic operations of the La-doped ZnO nanocomposites over five repeated cycles. The catalyst had a marginal decrease in MB photodegradation after the fifth run compared to the first cycle. The slight reduction in the catalyst could be related to the fractional loss or decline of the catalyst mass and the catalyst surface during the washing/drying procedure.

The photocatalyst activity was achieved for each new phase washed with deionized (DI) water and maintained the stability of other reaction conditions. After five cycles, no significant drop in the La_2_O_3_-ZnO photocatalytic degradation was observed. The photodegradation of MB and RhB was yet larger than 98%, suggesting that the nanocomposites of MB and RhB have considerable stability and excellent repeatability. The little reduction in the photocatalytic activity could be assigned to the surface toxicity of the intermediate catalyst, the limiting of the surface ability, and the velocity of the electron transfer.

## 4. Conclusions

In conclusion, the combustion approach successfully prepared ZnO doped with different concentrations of lanthanum oxides. From XRD and SEM analysis, the structural morphologies of the La_2_O_3_-ZnO nanocomposites were investigated, which revealed an upsurge in the grain size as the doping ratio of La_2_O_3_ increased. Additionally, UV-Vis optical diffuse reflectance (ODR) spectroscopy and Fourier transformation Infrared (FT-IR) spectroscopy were used to examine the optical characteristics of the La_2_O_3_-ZnO nanostructured materials. For the as-prepared La_2_O_3_-ZnO nanocomposites, the direct optical bandgaps range from 3.32 eV to 3.24 eV, and the indirect bandgaps are between 3.28 eV and 3.11 eV. That represented the catalyst’s absorption edge depended only on the ZnO nanostructure as a host matrix. In addition, the electrical behaviors of the as-prepared ZnO doped with lanthanum oxides at the nanoscale were investigated through current–voltage measurements and dialectical analysis for the ceramic varistors. The photocatalytic activity and degradation performance of the ZnO material are enhanced by doping a reasonable amount of lanthanum oxides. The proposed study reveals that the photocatalytic degradation performance improves as La_2_O_3_ load increases, indicating the promising photocatalytic performance of the synthesized La_2_O_3_-doped ZnO composites towards the degradation of MB and RhB dyes. S4 possesses the highest RhB and MB degradation, a maximum efficiency, in 30 min under visible light with 0.13 and 0.11 min−1 rate of reaction, respectively. The smart and multifunctional La_2_O_3_-ZnO nanostructures are intriguing for wide-scale technical, biomedical, and environmental applications, such as varistors, biosensors, and photocatalysis.

## Figures and Tables

**Table 1:** The sample code for La_2_O_3_-ZnO nanostructures doped with lanthanum ions at various concentrations.

Samples	Codes	La_2_O_3_/ZnO Molar Fraction
Pure ZnO	So	-------
0.001 g La_2_O_3_-ZnO	S1	0.00008
0.01 g La_2_O_3_-ZnO	S2	0.0008
0.1 g La_2_O_3_-ZnO	S3	0.008
0.5 g La_2_O_3_-ZnO	S4	0.041
1 g La_2_O_3_-ZnO	S5	0.080
2.5 g La_2_O_3_-ZnO	S6	0.179
5 g La_2_O_3_-ZnO	S7	0.304

**Table 2:** A comparison of the XRD-derived crystallite size, dislocation density, and lattice strain.

Samples	Phases	Mean Value of the Crystallite Size from XRD Analysis			Mean Values of the Grain Size from SEM Analysis, (nm)
		Crystallite Size (nm)	Dislocation Density, (nm)^2^	Lattice Strain	
So	Phase 1: ZnO	48.5162	0.00064	0.00083	108.86
	Phase 2: Carbon	20.1144	0.00361	0.00197	
S1	Phase 1: ZnO	46.5409	0.00066	0.00085	95.69
	Phase 2: Carbon	23.6019	0.00193	0.00151	
S2	phase 1: ZnO	30.0091	0.00155	0.00131	106
	Phase 2: La_2_O_3_	9.0647	0.01217	0.003824	
S3	Phase 1: ZnO	32.5562	0.00185	0.00139	91.87
	phase 2: La_2_O_3_	8.5231	0.01377	0.00407	
S4	Phase 1: ZnO	19.6393	0.00377	0.00204	88.64
	Phase 2: La_2_O_3_	18.1128	0.00305	0.00191	
S5	Phase 1: ZnO	22.0992	0.00239	0.00154	81.2
	Phase 2: La_2_O_3_	18.5326	0.00329	0.00195	
S6	Phase 1: ZnO	30.7257	0.00228	0.00141	114
	Phase 2: La_2_O_3_	30.6801	0.00183	0.00138	
	Phase 3: La_2_Zn	31.8144	0.00181	0.00135	
S7	Phase 1: ZnO	25.9268	0.00219	0.00152	138.82
	Phase 2: La_2_O_3_	30.1924	0.00171	0.00135	
	Phase 3: La_2_Zn	26.6642	0.002017	0.001457	

**Table 3:** Direct and indirect transitions of as-prepared La_2_O_3_-ZnO nanostructures.

Samples	*E_g_*_1_(d), eV	*E_g_*_1_(ind), eV
So	3.32	3.28
S1	3.24	3.11
S2	3.27	3.19
S3	3.30	3.18
S4	3.31	3.20
S5	3.26	3.15
S6	3.25	3.16
S7	3.28	3.17

**Table 4:** Reaction rate constants of MB and Rhodamine B degradation in the presence of all prepared samples.

Samples	Methylene Blue		Rhodamine B	
	K, (min^−1^)	Adj. R-Square	K, (min^−1^)	Adj. R-Square
**So**	0.0139	0.9943	0.0216	0.9870
**S1**	0.0174	0.9814	0.0239	0.9814
**S2**	0.0415	0.9928	0.0287	0.9928
**S3**	0.0512	0.9895	0.0437	0.9726
**S4**	0.1303	0.9593	0.1102	0.9249
**S5**	0.0680	0.9409	0.0607	0.9453
**S6**	0.05909	0.9726	0.0424	0.9726
**S7**	0.0414	0.9894	0.0401	0.9875

**Table 5:** Comparison of photocatalytic activities of some ZnO-based materials.

Photocatalyst	Doping %	Preparation Method	Organic Solution	Catalyst Load (g/L)	Irradiation Time	Source	% Degradation	Refs.
La-doped ZnO	0.8%	Co-precipitation	Monocrotophos	0.1	180 min	UV-light	0.0827 min^−1^ 97.3%	[62]
La-doped ZnO	0.8%	Co-precipitation	2,4,6-trichlorophenol	0.1	180 min	UV-light	0.032 min^−1^ 100%	[63]
La-doped ZnO	2%	Co-precipitation	RhB	0.1	200 min	UV-light	-----	[64]
La-doped ZnO	1%	Co-precipitation	MB	0.15	180 min	fluorescent lamp	0.0208	[61]
La-doped ZnO	0.5%	Co-precipitation	Metasystox	0.1	150 min	UV-light	90%	[65]
La-doped ZnO	3%	Gel-combustion	Paracetamol	0.1	180 min	Xe lamp	80%	[66]
La-doped ZnO	3%	microwave assisted sol–gel	MB	0.09	60 min	UV-light	95%	[67]
La-doped ZnO	1%	Co-precipitation	Paracetamol	0.1	180 min	Visible light	99%	[68]
La-doped ZnO	----	Co-precipitation	MB	0.01	100 min	Visible light	----	[69]
La-doped ZnO	1%	Co-precipitation	Congo-Red	0.1	240 min	UV-light	97.63%	[70]
La-doped ZnO	----	Gel combustion	MO	0.05	150 min	Visible light	85.86%	[70]
La-doped ZnO	0.5%	combustion	MB	0.01	80 min	Visible light	100%	Present work
La-doped ZnO	0.5%	combustion	RhB	0.01	80 min	Visible light	100%	Present work

## Data Availability

The data supporting this study’s findings are available from the corresponding author upon reasonable request.
